# Chronic Inflammatory Diseases, Anti-Inflammatory Agents and Their Delivery Nanosystems

**DOI:** 10.3390/pharmaceutics13010064

**Published:** 2021-01-06

**Authors:** Daniela Placha, Josef Jampilek

**Affiliations:** 1 Nanotechnology Centre, CEET, VSB—Technical University of Ostrava, 17. listopadu 2172/15, 708 00 Ostrava-Poruba, Czech Republic; 2 Centre ENET, CEET, VSB—Technical University of Ostrava, 17. listopadu 2172/15, 708 00 Ostrava-Poruba, Czech Republic; 3 Department of Analytical Chemistry, Faculty of Natural Sciences, Comenius University, Ilkovicova 6, 842 15 Bratislava, Slovakia; 4 Division of Biologically Active Complexes and Molecular Magnets, Regional Centre of Advanced Technologies and Materials, Czech Advanced Technology and Research Institute, Palacky University, Slechtitelu 27, 783 71 Olomouc, Czech Republic

**Keywords:** drug delivery systems, nanoformulations, nanoparticles, anti-inflammatory drugs, experimental drugs, inflammation

## Abstract

Inflammatory diseases, whether caused by excessive stress on certain tissues/parts of the body or arising from infections accompanying autoimmune or secondary diseases, have become a problem, especially in the Western world today. Whether these are inflammations of visceral organs, joints, bones, or the like, they are always a physiological reaction of the body, which always tries to eradicate noxious agents and restore tissue homeostasis. Unfortunately, this often results in damage, often irreversible, to the affected tissues. Nevertheless, these inflammatory reactions of the body are the results of excessive stress, strain, and the generally unhealthy environment, in which the people of Western civilization live. The pathophysiology and pathobiochemistry of inflammatory/autoimmune processes are being studied in deep detail, and pharmaceutical companies are constantly developing new drugs that modulate/suppress inflammatory responses and endogenous pro-inflammatory agents. In addition to new specifically targeted drugs for a variety of pro-inflammatory agents, a strategy can be found for the use of older drugs, which are formulated into special nanodrug delivery systems with targeted distribution and often modified release. This contribution summarizes the current state of research and development of nanoformulated anti-inflammatory agents from both conventional drug classes and experimental drugs or dietary supplements used to alleviate inflammatory reactions.

## 1. Introduction

Diseases for the treatment of which the use of drug delivery nanosystems (nanoDDSs) for anti-inflammatory drugs is considered are the result of chronic inflammation or autoimmune diseases (e.g., allergies, atopic dermatitis, psoriasis, asthma, chronic obstructive pulmonary disease, arthritis (osteoarthritis, rheumatoid arthritis), inflammatory bowel diseases (ulcerative colitis, Crohn’s disease), celiac disease, autoinflammatory syndrome, or inflammation accompanying transplant rejection). Chronic inflammatory diseases are one of the most common reductions in quality of life and are among the most common causes of death. This is mainly a problem of Western countries associated with the way of life, stress, and environmental burden. For example, the prevalence of chronic inflammatory diseases is expected to increase steadily in the United States over the next 30 years. According to the research by Rand Corporation, in 2014, nearly 60% of Americans had at least one chronic condition, 42% had more than one, and 12% of adults had five or more chronic conditions [1,2,3,4,5,6]. Inflammatory bowel disease (IBD) was diagnosed in approx. 1.3% of adults in the USA in 2015. These IBD positives are also more likely to develop other chronic diseases such as cardiovascular, respiratory diseases, cancer, arthritis, or kidney and liver diseases [7]. Rheumatoid arthritis (RA), lupus erythematosus, gout, or fibromyalgia were diagnosed from 54.4 million adults in the United States (22.7%) between 2013 and 2015. RA has a global prevalence of around 1% with the incidence in women being 2–3 fold more than in men. In addition, RA is associated with an increased risk of internal organ damage and systemic complications, with premature atherosclerosis being the most important [8,9,10,11]. According to the WHO, three out of five people worldwide die from diseases associated with chronic inflammatory diseases such as stroke, chronic respiratory diseases, heart disorders, obesity, and diabetes. Although atherosclerosis, glomerulonephritis, hepatitis, diabetes, or various cardiovascular diseases do not appear to be diseases associated with inflammation, they can be included among diseases caused by chronic inflammation or where chronic inflammation is involved in progression [1].

Inflammation is a complex, stereotypical reaction of an organism to damaged cells and organs and vascularized tissues. In principle, inflammations can be distinguished into: (*i*) defensive (defensive-adaptive or reparative) and (*ii*) harmful. Depending on the causative agent and the extent of the damage, the inflammatory response may proceed very rapidly or may develop into a complex process involving many cell types. These interact with each other through special adhesive molecules and receptors. In this case, the synthesis of various cytokines, growth, transformation, chemotactic, cytotoxic, and other factors that regulate the course of the inflammatory reaction is induced in them. It is also affected by vasoactive mediators of endothelial and other cells, icosanoids, reactive oxygen species (ROS), products of multienzyme systems of blood plasma (complement, hemocoagulation, fibrinolytic, kinin), various hormones, neurotransmitters, and neuropeptides. Many of them apply not only in defensive, but also in damaging processes that are part of the pathogenesis of many diseases [12,13,14].

The main task of inflammation is to deal with the damage or cause corrective measures aimed at restoring homeostasis and survival of the organism. In doing so, it is important to initially transfer fluid, proteins, and cells from the bloodstream to the damaged tissues. This is made possible by the following mechanisms: (*i*) vasodilation (increased blood flow in the affected area); (*ii*) increased vascular permeability (allows diffusible components to reach the site of inflammation); (*iii*) cell infiltration (migration of inflammatory cells across the vessel wall to the site of injury, controlled by chemotactic factors, cytokines, and adhesive molecules); (*iv*) changes in the biosynthetic and metabolic profiles of cells and organs; and (*v*) activation of the immune system and blood plasma enzyme systems [12,13,15].

Inflammation is manifested by redness, heat, swelling, pain, and dysfunction of the affected tissue. The most important features include edema formation, fibrin deposition, and the presence of neutrophils in the injured tissue. Depending on the nature of the stroke or the extent of the injury, the neutrophil count may decrease, leading to the development of chronic inflammation characterized by an increased number of macrophages, lymphocytes, plasma cells, and eosinophils. Three situations can arise at this stage: (*i*) the pathogen is eliminated and the injured tissue is repaired to regain normal structure and function; (*ii*) the pathogen cannot be eliminated, it remains at the site of damage and concomitant activation of immune mechanisms leads to the formation of granulomatous or damaging inflammation; or (*iii*) the tissue injury is irreversible, resulting in scarring and loss of original function [13,15,16].

The development and course of inflammatory reactions are regulated by cytokines (released mainly from leukocytes), products of blood plasma enzyme systems, lipid mediators—icosanoids (prostanoids, leukotrienes, thromboxanes, platelet activating factors—PAF) secreted from various cells, and vasoactive mediators released from endothelial and mast cells, basophils, and platelets. Inflammatory mediators regulating various types of reactions are different. In inflammatory reactions initiated by immune mechanisms, the antigen itself has a key regulatory function. For this reason, the accumulation of cells in an autoimmune reaction is similar to that at a site of chronic infection (i.e., antigen cannot be removed) [15,16,17,18].

For acute inflammation, two phases are typical: (*i*) acute vascular response that occurs within a few seconds after injury and lasts for several days, manifested by vasodilation, increased vascular permeability, resulting in hyperemia, erythema, and edema; and (*ii*) acute cellular response that occurs in the case of severe damage within a few hours; granulocytes (especially neutrophils) and erythrocytes appear; fibrin is deposited in the damaged site; clots form; the dead cells give rise to pus, which also contains leukocytes or bacteria [15,19].

A chronic cellular response may occur in the following days if the damage is severe enough. Mononuclear cells (macrophages and lymphocytes) appear in the deposit, the task of which is to kill penetrating microorganisms, capture and absorb cell and tissue debris, and participate in healing and tissue remodeling. Chronic inflammation is associated with damage to tissues and their components. Pathogenetically, it is a long-term process, in which destruction and inflammation are accompanied by attempts of the body to heal. The beginnings of chronic inflammation can be completely inconspicuous and, thus, overlooked. They can be divided into (*i*) chronic inflammation as a consequence of acute inflammation, or (*ii*) chronic inflammation arising de novo [15,19,20].

All factors causing acute inflammation as well as immune hypersensitivity reactions, autoimmunity, and immunocomplexes with the key involvement of T-lymphocytes, neutrophils, and some cytokines (tumor necrosis factor (TNF), interleukin (IL)-1, IL-15) can be the etiological cause of chronic inflammation. Neutrophils, Th1-lymphocytes, and macrophages are mainly involved in tissue damage in chronic inflammation [13,16,18].

Neutrophil damage consists of premature activation, excessive release of toxic products, and, together with macrophages, loss of ability to terminate the inflammatory response. In the physiological course of the inflammatory reaction, healing—the restoration of normal function and tissue architecture—occurs within a few days to weeks. Precipitates are removed by fibrinolysis and, if this is not possible, in order for the tissue to regain its original form, a scar is formed, which is formed by fibroblasts, new collagen, and new endothelial cells [18,19,20].

Significant is the formation of inflammatory infiltrate (exudate), which has liquid and cellular components. All components of blood plasma including fibrinogen, immunoglobulins, complement, kinins, etc., are in liquid. Cell exudate is formed in acute and chronic cellular responses (second and third stages of inflammation). Initially, neutrophils predominate in it, later mononuclear cells (macrophages, lymphocytes). Neutrophils have a central effector function in acute inflammation. Mononuclear phagocytes are the major exudate cells in the subacute and chronic phases. Eosinophils and basophils predominate when inflammation is initiated by an anaphylactic reaction or parasites. Specific phagocytes (neutrophils, eosinophils, monocytes, and macrophages) phagocytose foreign material; lymphocytes are the key cells of the immune response; endothelial cells regulate the transfer of leukocytes from blood to inflammatory tissue, and platelets with mast cells produce mediators of the early phase of inflammation [16,18,19,20].

Inflammatory mediators are soluble diffusible molecules that are released from inflammatory or other cells. They act both locally at the site of tissue damage and infection and at anatomically distant sites. The mediators are divided into: (*i*) exogenous: (mainly bacterial products and toxins (e.g., lipopolysaccharides of Gram-negative bacteria); and (*ii*) endogenous (arise from the activity of the immune, complement, hemocoagulation, fibrinolytic, and kinin systems and regulate inflammation and homeostasis; components of these systems are physiologically inactive in plasma, and after activation, they are released in the form of active peptide fragments. Inflammation mediators are released at the site of tissue damage from various cells, in which they are either present as preformed molecules in granules (histamine) or, if necessary, rapidly synthesized and extracellularly released (metabolites of arachidonic acid) [13,16,18,19].

Early phase mediators are important in acute inflammation. These include histamine, serotonin, vasodilators (NO, PGI2, PGE2, PGD2), vasoconstrictors (endothelins, TXA2, PGG2, PGH2), vascular endothelial products, chemotactic factors (C5a), and cytokines (IL-1, IL-6, TNF). Late phase mediators are responsible for regulating vascular reactivity within 6–12 h after the initiation of the inflammatory response and for transporting leukocytes into the tissue. Arachidonic acid metabolites are involved in the regulation of vascular reactivity. The formation of edema is caused by vasodilators: histamine, bradykinin, NO, PGI2, PGE2, C5a, and C3a. This process is associated with the release of chemotactic factors LTB4, PAF, *N*-formylmethionyl peptides from bacteria, and mitochondria of damaged host cells, which directly stimulate the migration of specific phagocytes [13,16,18,19].

## 2. Drugs with Anti-Inflammatory Effect

**Figure 1:**
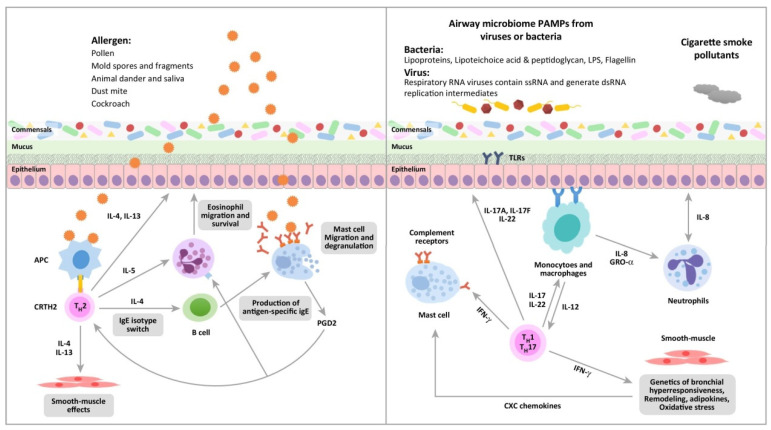
The diagram shows potential triggers and innate immune response of eosinophils. Respiratory infections caused by bacterial and viral infections activate structural and immune cells, cause inflammation, and affect reactions to other allergens, pathogens, and pollution. Adapted from [22], Elsevier, 2015.

As demonstrated above, the inflammatory process is a complex matter and especially chronic inflammations have different origins. In addition to “simple inflammation” caused by an injury, inflammatory diseases of visceral organs, joints, bones, etc. can be based on an infectious or autoimmune basis, with the body always trying to eradicate harmful substances and restore tissue homeostasis [12,21]. Bacterial infections are often associated with serious inflammatory diseases such as bronchial asthma [22,23,24], gingivitis, periodontitis [25], systemic lupus erythematosus [26], and even cancer [27]. As bacterial infections can cause inflammation, which can cause consequent damage to surrounding tissue [16,18,28], anti-inflammatory drugs are very often combined with drugs that have antimicrobials effects, at least for the first time to control an attack (Figure 1).

These combinations can be realized most simply as the simultaneous administration of two or more drugs in several formulations or the administration of so-called combo preparations (i.e., fixed combinations (frequently two active ingredients in one formulation) or so-called codrugs (where the final molecule consists of two different drugs linked by a degradable bond) [29,30,31]). Another approach, which is based on the so-called multi-target agents, aims to develop drugs with dual anti-inflammatory and antimicrobial activity [32]. This approach is based on the concepts of privileged scaffolding, polypharmacology, and multifactorial diseases [33,34,35,36,37,38,39]. Thus, multi-target drugs can be designed for the simultaneous treatment of, for example, autoimmune, inflammatory, and invasive diseases. From a pharmacoeconomic point of view and patient comfort, it seems advantageous to treat both cause and effect (bacterial infection and inflammation) simultaneously with one active ingredient [30,40].

However, if attention is paid to conventional anti-inflammatory drugs, they can be divided according to the mechanism of action and according to the structure. The classic “universal” anti-inflammatory drugs are glucocorticoids and cyclooxygenase (COX 1 and 2) inhibitors. Thus, both groups are able to intervene in inflammation by affecting the metabolism of arachidonic acid. In addition to these two basic classes, other drugs that are used to treat specific chronic inflammatory diseases such as asthma, COPD, psoriasis, RA, IBD, etc. can be found [1,2,41].

Combination therapy is used to treat asthma and COPD, except for inhaled glucocorticoids (e.g., beclomethasone, budesonide, fluticasone, flunisolide, ciclesonide) and inhaled (fenoterol, pirbuterol, salbutamol, terbutaline, formoterol, salmeterol) or p.o. (clenbuterol, prokaterol, bambuterol) β_2_-mimetics, long-acting theophylline- methylxanthines (theophylline, aminophylline, etophylline), long-acting anticholinergic agent tiotropium bromide, long-acting inhibitor of the enzyme phosphodiesterase-4 (roflumilast), cromones (cromoglycate, nedocromil), and other cytokine and histamine release inhibitors (tranilast) or antileukotrienes (montelukast) may be alternatively used [41,42,43,44].

The treatment of psoriasis consists of the topical application of ichtamol, dithranol, salicylic acid, urea, and/or corticosteroids (mometasone, budesonide) or, in moderate and severe cases, fluorouracil or methotrexate (antimetabolites) in ointment, selective vitamin A derivatives—retinoids (acitretin, adapalene, tazarotene), and vitamin D_3_ analogues (calcitriol, calcipotriol, tacalcitol, oxacalcitriol, paricalcitol). Systemic therapy is indicated for generalized disease states and consists mainly in the application of cytostatics (6-thioguanine, azathioprine, hydroxyurea, methotrexate, fumaric acid esters, mycophenolic acid) and immunosuppressants (cyclosporin A, tacrolimus, pimecrolimus) [41,43,44,45].

The optimal treatment for IBD is based on a suitable diet. Overall, the diet should be residue-free, non-irritating, sufficiently high in calories, and balanced with plenty of vitamins (especially B-series) and minerals (calcium, iron, magnesium, zinc). In acute conditions, it is important to reduce fiber and caffeine. Probiotics have been used successfully recently, because research has suggested that the microflora as a major antigenic stimulus is involved in provoking the disease in genetically predisposed individuals [46,47,48,49]. In acute conditions, glucocorticoids (hydrocortisone, prednisolone, methylprednisolone, triamcinolone, budesonide) are indicated in maintenance therapy, other drugs: aminosalicylates (sulfasalazine, mesalazine, sulfapyridine, 5-aminosalicylic acid), immunosuppressants/cytostatics (cyclosporin A, tacrolimus, azathioprine, 6-mercaptopurine, methotrexate), and antibacterial chemotherapeutics (metronidazole, ornidazole, ofloxacin, ciprofloxacin). Agents for this “conservative” treatment are administered in the form of enemas, suppositories, and enteric tablets, or, in severe conditions, systemically. As supportive and symptomatic therapy, treatment with antidiarrheal drugs (diphenoxylate, loperamide), spasmoanalgesics, anxiolytics, or antidepressants in patients with an unfavorable course or a major mental superstructure are indicated [41,43,44,50,51].

**Scheme 1:**
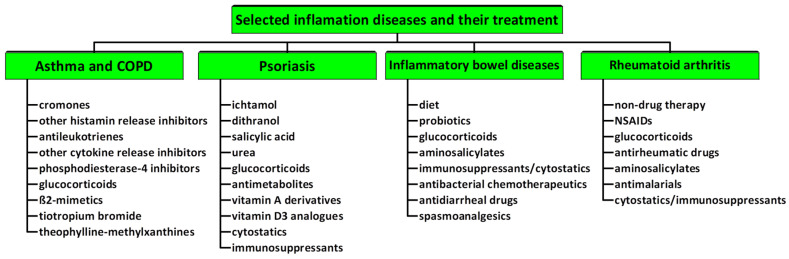
Summary of described inflammation diseases and their treatment.

The main goal of RA treatment is to induce the remission of the disease, or suppression of inflammation, reduction of pain, maintenance of muscle strength, maintenance of function, improvement of quality of life, maintenance of work ability, and suppression of joint destruction. Therapies are divided into: (i) non-drug (rehabilitation, physiatry, balneology), and (ii) drug, which consists of the administration of general anti-inflammatory drugs NSAIDs (indomethacin, diclofenac, ibuprofen, piroxicam, tenoxicam, meloxicam, nimesulide, celecoxib, parecoxib, lumiracoxib), glucocorticoids (hydrocortisone, prednisolone, methylprednisolone, triamcinolone, dexamethasone, betamethasone) and then the administration of so-called disease-modifying antirheumatic agents: antirheumatic drugs (auranofin, aurothiomalate, d-penicillamine), aminosalicylates (sulfasalazine), antimalarials (chloroquine, hydroxychloroquine), and cytostatics/immunosuppressants (methotrexate, cyclophosphamide, azathioprine, minocycline, leflunomide, cyclosporin A, tacrolimus) [41,43,44,52]. The summary of the drugs used for the above named inflammatory diseases treatment are listed in Scheme 1.

Biological therapy is a new category of therapy for most inflammatory/autoimmune diseases. It uses antibodies that neutralize proteins that cause inflammation in the body. They are given by infusion or injection. These include, for example, infliximab, etanercept, adalimumab, anakinra, vedolizumab, golimumab, tocilizumab, ustekinumab, certolizumab, etc. [53,54].

In addition to the special drugs/medicinal procedures above-mentioned, it has been observed that other drugs traditionally used in the treatment of other diseases are able to suppress chronic inflammation; an example is the antidiabetic drug metformin or antihyperlidemic drugs from the group of statins [1,2,41].

In addition to drugs, there are many natural agents and nutritional supplements that can be used as additives to reduce inflammation. These are mainly fish oil, lipoic acid, sesame oil, and curcumin. Among other things, they bring benefits in the treatment of cancer and heart disease. In addition, some herbal supplements such as ginger, cinnamon, garlic, cayenne, cannabis, hyssop, and *Harpagophytum procumbens* can also help alleviate chronic inflammatory diseases [1,47,55,56,57].

Lifestyle adjustments can also be helpful in combating chronic inflammation. The most effective is getting rid of chronic stress, quitting smoking and drinking alcohol, weight loss, and diet modification (i.e., introducing low-glycemic diets, reducing the intake of saturated fats and *trans*-fats, increasing the intake of omega-3 polyunsaturated acids, and increasing the intake of fresh fruits and vegetables with a high content of natural antioxidants and polyphenols). Vegetables also contain a lot of fiber, the intake of which is associated with a reduction in the level of pro-inflammatory agents such as ILs and TNF. Consuming nuts and mungo and drinking green and black tea also have benefits. It is also advantageous to maintain higher levels of vitamins (especially D and E) and microelements such as magnesium, zinc, and selenium [1,47,56,57].

## 3. Drug Delivery Systems

NanoDDSs represent a fast-developing scientific field where nano-scale materials (generally defined in dimensions of 1–100 nm) can be used as diagnostic tools or for the transfer of therapeutic agents to specific targeted sites in an organized way [58,59,60]. In nanomedicine, the size of nanoparticles (NPs) has increased to 500 nm and even to 1000 nm due to the ability of nanoparticles to change the properties of the drug induced by their dimensions with a still high enough surface-to-volume ratio [58,61,62]. Many excellent biological, chemotherapeutic, or immunotherapeutic agents have been proposed in the treatment of various diseases, and multiple benefits in treating chronic diseases by site-specific and target-oriented delivery of precise medicines were confirmed in a number of research works [58,59,60].

**Figure 2:**
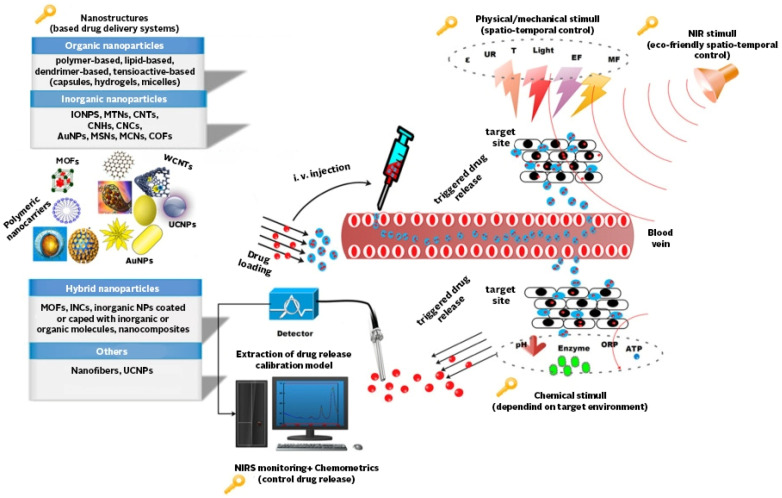
Scheme of basic nanostructures for DDS and different stimulation. Adapted with permission from [60], Elsevier, 2017.

The non-selective availability of drugs accompanied by the need to use potentially too high doses belong to common problems and limitations in traditional therapy. This therapeutic disadvantage can be overcome by DDSs that address problems with drug stability, solubility, and permeability as major causes of the damage to the normal cells and an increase in the intensity of harmful side effects. The use of suitable DDSs allows overcoming those problems and ensuring the transfer of a drug to the infected site with consequent release under selected conditions [60,63,64,65,66]. The use of nano-scale drug carriers appears to increase drug specificity and leads to a reduction in adverse effects due to the reduced drug dose [67,68,69,70,71,72,73,74,75,76,77,78] (see Figure 2).

Initially, nanomaterials in drug delivery systems were used to shield the drugs and transport them to the diseased tissue or organ. The next generation was focused on targeting the diagnostic material or drug to the diseased cell-specific receptors or to a particular organ. The latest generation of nanotransporters are the stimuli-responsive nanocarriers that are based on the exploitation of disease condition or environment to develop a system with the best diagnosis and treatment effects [65]. Several possibilities of stimuli-responsive nanosystems such as pH, temperature, redox state, or magnetic field based systems have been tested with promising efficiency [60,65,79,80,81,82,83].

NanoDDS are widely investigated for cancer therapy, cardiovascular diseases treatment, anemia, and hemophilia treatment, in nutraceutical delivery, and also for neurodegenerative, infectious, autoimmune, ocular, and pulmonary diseases as well as for diagnostic, regenerative therapy, and other purposes [69,70,71,72,73,74,75,76,77,84,85].

As above-mentioned, inflammation accompanying chronic injury and autoimmune diseases is a starting point leading to tissue dysfunction and degeneration. Inflammatory monocytes and neutrophils enter inflamed tissue, and common pharmaceuticals have not substantially improved outcomes also showing side effects [86]. The specific uptake of nanoparticles by immune-specific receptors in inflamed barriers is an example of a selective drug delivery to the inflamed tissue [67]. Advances in nanotechnologies and research into the pathogenesis of immune-mediated diseases allow for the development of new therapies using nanoparticles. Previous discoveries of interactions of nanoparticles with the mononuclear phagocyte system and ways how they affect its function during homeostasis and inflammation have emphasized the potential of nanoparticle-based therapies to control severe inflammation and restore peripheral immune tolerance in autoimmune diseases [87].

Generally, a DDS consists of three parts: the substrate (the drug carrier), the drug, and the stimuli-responsive agents. In nanoDDSs, the substrate is characterized by high loading capacity, low toxicity, and also by the ability to impregnate supramolecules on its surface [60]. These properties are determined by the entrapment efficiency, the drug loading efficiency, and the drug loading content. The impregnation of supramolecules is based on the presence of a supramolecular organic molecule, for example, rotaxane, which consists of a long chain-like molecule passed through a cyclic molecule. By attaching bulky “blocking” molecules, the cyclic molecule cannot slip and is thus held in place by mechanical bonds. Under certain conditions, the cyclic molecule may be more attached to one end of the rotaxane and may move to the other end in the presence of a stimulus. Combining one end of this organic structure with mesoporous silica nanoparticles has opened up wide possibilities for DDSs [88].

A number of different types of nanocarriers such as liposomes, dendrimers, polymeric nanoparticles (micelles, spheres, capsules), polymeric complex nanoparticles, cyclodextrins, nano-caseins, nanocrystals, electrospun nano-fibers, electro-sprayed nano-particles, nano-spray dried particles, covalent organic frameworks, hydrogels, inorganic nanoparticles (silica, iron, gold, titanium, carbon based nanoparticles) as well as hybrid nanoparticles (organic-inorganic based structures) have been developed. The research is focused on their in vivo absorption, permeation, and release together with toxicity, residual solvents, and biological fate during digestion, absorption, and excretion [59,60,82,83,89,90,91,92].

Drugs can be linked to the substrate via physical, electrostatic, or covalent bonding. Covalent bonds may prevent release before reaching the target site and reduce the drug leakage [60,93]. The stimuli-responsive agents can stimulate the release of a drug under endogenous or exogenous conditions localized at the target site [60]. The stimulation can be performed via chemical (endogenous conditions), physical, or mechanical ways or via a combination of chemical and physical ways. In the case of a chemical principle, the drug is released from the carrier due to the drug diffusion affected by intrinsic conditions such as the pH in the environment of the targeted cells or by the enzymatic cleavage, the hydrolyze, the ionic strength, and redox gradient. The physical principle of the drug release involves the effects of the magnetic or electric field, light, and temperature. Mechanical stimuli for the drug release are ultrasound, pressure, shear, strain (compression, stretching, bending), tension, and others. Combinations of the chemical and physical principles are proposed if the external physical stimuli is not able to pervade to the treated tissue. The combination of the electric field and pH, temperature and the electric field, the magnetic field and light in NIR, the redox conditions together with pH stimuli and many others have been described [59,60,82,83,94].

**Figure 3:**
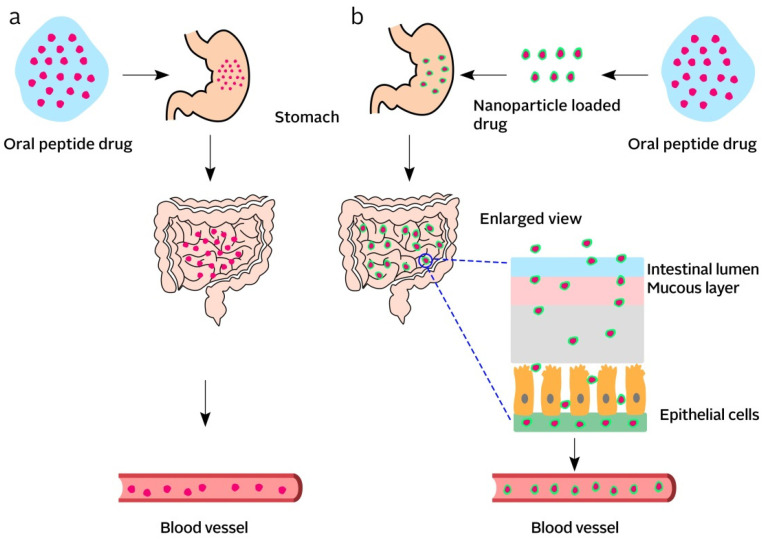
Oral administration of pH sensitive peptide drug: (**a**) an orally administered peptide drug is degraded particularly in the stomach due to proteolytic enzymes, which results in poor drug availability, (**b**) nanoparticles protect drugs and prevent them from enzymatic degradation. The drug distribution is more efficient. Adapted with permission from [89], Elsevier, 2019.

DDSs can be administered as oral DDSs, intravenous DDSs, subcutaneous DDSs, or inhalation DDSs [93]. The basic characteristics of all these nanosystems including aerodynamic diameter, density, biodegradability time, and bioadhesive properties need to be taken into account as they significantly affect the bioavailability of transported drugs. The most preferable mode of drug administration is the oral route; however, efficient drug delivery is limited by many side effects and several physiological barriers such as various cell types in the gastrointestinal tract, the presence of mucus that differs in thickness and structure, and differences in pH values and enzymatic degradation [95]. Nanoparticles exhibit exclusive properties such as small size and high surface area, which can be modified in various ways and tailored to achieve specific effects. Nanoparticle formulations can enhance the stability of a drug in the environment of the gastrointestinal tract. They can be modified to target specific sites in the tract; they increase bioavailability and solubility of the drug and provide sustained release in the gastrointestinal tract [96,97]. The oral nano DDSs are absorbed into the blood differently compared to free drugs in traditional formulations with the main contribution of lymph absorption and endocytosis of nanoparticles [98]. Intravenous or oral DDSs are suitable for the administration of peptides and proteins because they are protected against degradation by proteolytic enzymes in this way (Figure 3). The intravenous DDSs act quickly and ensure drug bioavailability even in lower doses. Subcutaneous DDSs are administered in the form of nanoemulsions, vesicles (niosomes, ethosomes, liposomes), or nanoparticles. They enter the skin through the intercellular (through lipid matrix filling the intercellular spaces of the keratinocytes), transcellular (through keratinocytes), or the transappendageal pathways (across hair follicles, sweat glands, or sebaceous glands). For lung disease treatment, the inhalation of a nanoDDS system can be used. Drugs incorporated in nanoparticles are inhaled, they pass through the oropharynx, and they are deposited in the lung alveoli. Then, the drug is permanently released from the lungs and distributed in systemic circulation [89].

The great significance belongs to the research of the nanotoxicity of nanomaterials used in nanoDDSs. The unique properties of nanomaterials and nanoparticles make them very promising in biomedical applications, but some of their properties can make them toxic and risky for living organisms. For example, anionic nanoparticles are less toxic then cationic nanoparticles. The latter can induce ROS or homeostasis disruption or be accumulated in organs [89,99,100].

## 4. Designed NanoDDSs

### 4.1. NanoDDSs Developed for Selected Autoimmune Diseases

IBD including Crohn’s disease and ulcerative colitis, characterized by chronic recurrent gastrointestinal inflammation, is treated by delivering orally administered drugs to the colon because it improves drug efficacy and reduces systemic toxicity. However, targeting oral drugs to the colon, which is located at the distal end of the gastrointestinal tract, is difficult due to physiological challenges and biochemical and environmental barriers including mucus and epithelial barriers [101]. Thus, conventional colon drug delivery systems have improved the treatment of IBD, but therapy often leads to inconsistent problems with efficacy and toxicity. New nanoparticle-based approaches offer several advantages over conventional dosage forms due to their ability to selectively target inflamed tissues. Various mechanisms/strategies including size-, charge-, pH-, pressure-, degradation-, ligand-receptor-, and microbiome-dependent drug delivery systems have been exploited in preclinical studies. A certain number of NP delivery systems have sought to target drugs to the inflamed intestine. Although several NP-based drugs have entered clinical trials for the treatment of IBD, most have failed due to premature drug release, weak targeting ability, and the high immune toxicity of some of the synthetic nanomaterials that have been used to fabricate the NPs [102,103,104,105,106]. Additionally, numerous in vitro and in vivo experiments have demonstrated that not only nanoscaled synthetic drugs, but also phytochemicals and macromolecules encapsulated in nanoparticles can be used for the treatment of IBD and IBD-associated colorectal cancer [51,107,108].

Likewise, periodontitis is a chronic inflammatory disease of the periodontal tissues caused by pathogenic microorganisms and characterized by the disruption of tooth-supporting structures. Conventional administration of drugs for the treatment of periodontal disease encounters poor biodistribution, low selectivity of action, rapid drug release, and damage to healthy cells. To overcome these limitations, controlled drug delivery systems are being developed as a method of treating oral infectious diseases including periodontitis. Various polymer-based and lipid-based delivery systems are being developed such as polymer–drug conjugates, dendrimers, polymeric micelles, nanocapsules, nanospheres, hydrogels, and liposomes [109,110].

The unique structure of bone and cartilage makes the systemic delivery of free drugs to those connective tissues very challenging. Consequently, effective and targeted delivery for bone and cartilage is of utmost importance. Biodegradable polymers enable designing carriers for a targeted and temporal controlled release of one or more drugs in concentrations within the therapeutic range. Furthermore, tissue engineering strategies can allow drug delivery to advantageously promote the in situ tissue repair. Thus, various DDSs based on biodegradable biomaterials have been developed for the treatment of not only osteoporosis and inflammatory arthritis (osteoarthritis and rheumatoid arthritis), but also cancer and, in addition, for the tissue engineering of bones and cartilage. These designed DDSs have high added value, but some problems persist and are mainly related to an appropriate residence time and a controlled and sustained release of the therapeutic agents over a prolonged period of time [111,112,113]. Osteoarthritis is characterized by pathological changes in joint tissues and cells. The joint is a difficult area for drug administration, because the joint has poor bioavailability for systemically administered drugs and therapeutics disappear rapidly after intra-articular injection. In addition, each tissue in the joint presents unique barriers to drug localization. Nanomaterials have reliably demonstrated improved drug retention profiles in the common space compared to injecting bulk drugs. In addition, nanomaterials have been engineered through active and passive targeting strategies to facilitate interactions and localizations in specific joint tissues such as cartilage and synovium [114]. The conventional drug therapy of RA has many disadvantages like low bioavailability, rapid metabolism, poor absorption, first-pass effect, and serious adverse effects; therefore, the utilization of these drugs through the oral and parenteral route is limited. Therefore, a carrier system is required that should deliver the drug to the target site with minimal side effects. In this connection, nanocarrier systems are of prime importance because of the associated benefits such as their nano-scaled size, targeted drug delivery, and reduced toxicity that can improve the patient’s compliance. Novel DDSs like microspheres, nanoparticles, dendrimers, liposomes, etc. seem to be promising tools in overcoming the disadvantages. Thus, nanoDDSs involving polymers and hydrogels were investigated. In addition, nanocarrier systems based on ceramics like hydroxyapatite have gathered striking attention due to their bioactive, biocompatible, and bio-conductive characteristics. Nano-sized hydroxyapatite (HA) permeates the bone tissues and serves as a source of calcium phosphates required for repairing bones that are damaged during disease process. Moreover, transdermal delivery systems of nanonized drugs seem to be useful strategies to improve problems with drug delivery for the treatment of RA [10,115,116,117,118].

### 4.2. NanoDDSs for Drugs

Polymeric NPs composed of dexamethasone (DEX) and model antigen ovalbumin (OVA) in a poly(lactic-*co*-glycolic acid) (PLGA) matrix were prepared by the water-in-oil-in-water double emulsion solvent evaporation method. It was confirmed that immature dendritic cells (DCs) treated in vitro with the nanosystem did not mature into immunogenic DCs, but instead were converted into tolerogenic DCs. In addition, the decreased production of OVA-specific cytotoxic T cells and OVA-specific IgG production in mice and an increase in regulatory T cells were found. Mice fed by these NPs showed OVA-specific immune tolerance. These results suggest that such a system combining antigen with DEX without systemic effects can be used to develop antigen-specific immune tolerance, which is essential for the treatment of autoimmune diseases [119]. Zhang et al. prepared a hydrogel consisting of ascorbyl palmitate, which contained DEX as the active ingredient [120]. The formulation was stable, and DEX release occurred only after enzymatic cleavage. Adhesion to inflamed epithelial surfaces has also been demonstrated in vitro and in mouse models of colitis in vivo. Overall, the system led to a significant reduction in inflammation at lower maximum systemic concentrations of DEX, indicating that the system appears to be promising for targeted enema therapies in patients with IBD colon. Assali et al. prepared a nanoformulation of DEX and diclofenac encapsulated in polylactide NPs to improve their solubility and create a sustained release system. In vitro release showed a sustained release profile of drugs up to 52 h. Anti-inflammatory activity was evaluated in BALB/c mice, and the expected synergistic effect (higher inhibition of TNF-α) was found compared to the individual drugs after 6 h of treatment [121].

Date et al. focused on the development of a budosonide (BUD) nanosuspension with approx. 200 nm particle size, composed of polystyrene coated with Pluronic^®^ F127, which is inert to mucosa and provides good mucosal distribution and BUD penetration into tissues in trinitrobenzenesulfonic acid-induced IBD mice. This IBD model demonstrated that daily treatment with an NS budesonide enema resulted in a significant reduction in macroscopic and microscopic IBD manifestations compared to untreated controls or mice treated daily with micronized budesonide (Entocort^®^) [122].

Methylprednisolone and bovine serum albumin (ME BSA NP) nanoparticles prepared by chemical crosslinking with the particle size of 131.1 ± 3.4 nm, the PDI of 0.159 ± 0.036 and the capture efficiency of 71.51 ± 1.74% were administered to rats with induced membrane glomerulonephritis (MGN). The results showed significantly reduced levels of 24-h urinary protein and serum creatinine, leading to the hypothesis of the potential use of this system for drug delivery for MGN and other complex chronic inflammation [123].

A new ophthalmic nanoparticulate gel composed of chitosan (CS), sodium deoxycholate (SD), and prednisolone acetate (PA) was prepared by Hanafy et al. with the particle size of 480 nm ± 28 and the PDI of 1.396. In simulated tears of pH 7.4, the release of PA was found to be 98.6% in 24 h. The results of the anti-inflammatory in vivo test of the gel with NPs on guinea pig eyes was significantly better than the effect of the gel loaded with micronized PA [124].

Various drugs from the NSAID class were encapsulated in biodegradable nanocrystalline cellulose (NCC) extracted from *Citrus limetta albedo*, which was bleached and hydrolyzed with sulfuric acid to prepare particle sizes ranging from 1 to 10 nm. Then, the NCC surface was modified with the cationic surfactant cetyltrimethylammonium bromide, which resulted in increases in surface area and encapsulation capacity. As a result, the carrier was able to function as a sustained release nanoDDS for 3 h [125]. SLNs composed of Capmul^®^ GMS-50K and Gelucire^®^ 50/13 encapsulating ibuprofen (IBP), ketoprofen (KTP), and nabumetone (NBT) were synthesized to allow controlled drug release and to prolong their plasma half-life and reduce side effects. The encapsulation efficiencies (EEs) of 53%, 74%, and 69% were found for IBP, KTP, and NBT, respectively. Slow, stable, and sustained drug release was observed for more than six days and the prepared SLNs were not toxic to the cell line [126]. Shah et al. incorporated indomethacin (IND), KTP, and nimesulide into stearic acid-based SLNs prepared using a microwave-assisted microemulsion. The NPs had a small particle size distribution, a negative zeta potential, and a high EE. Due to the core-shell structure of the SLN (drug-containing shell), biphasic drug release from the SLN was observed. The system showed dose-dependent cytotoxicity against the A549 cell line and suppressed the secretion of IL-6 and IL-8 in lipopolysaccharide-induced cells. All the said nanoDDSs appear to be promising for the further development of topically, orally, and/or nasally administered formulations [127].

Lipoid nanoDDS loaded with naproxen (NPX) with EE 99.8% designed for intra-articular administration was tested in a rat model of acute inflammation of the temporomandibular joint. In vivo results showed that the sustained delivery of NPX directly to the affected joint decreased leukocyte and pro-inflammatory IL-1β and TNF-α migration for more than a week, making this nanoformulation a promising candidate for the safe treatment of arthritic states [128].

**Figure 4:**
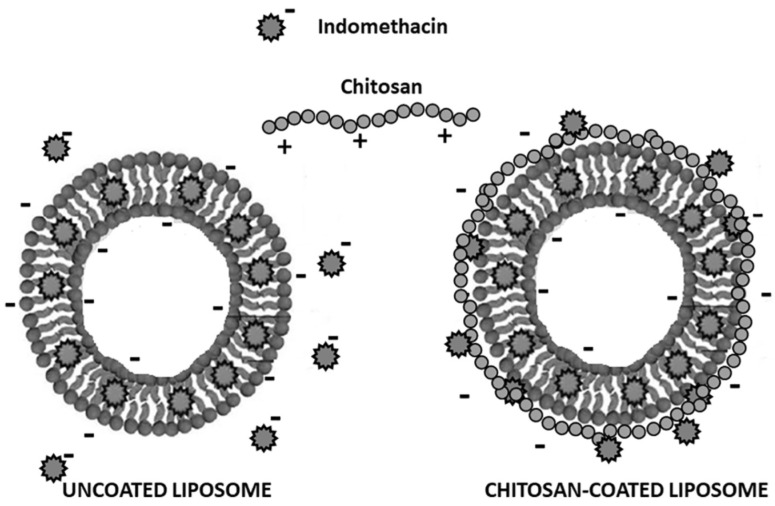
Scheme of structures of uncoated liposome and chitosan-coated liposome vesicles. Adapted with permission from [129], Elsevier, 2018.

Hybrid CS-coated nanoliposomes encapsulating IND (Figure 4) with 99% EE were prepared using a new simil-microfluidic method by Dalmoro et al. [129]. The system was tested for its mucoadhesiveness and a gastro retentive behavior in simulated gastric and intestinal fluids and has been found to be a suitable nanoDDS for the oral controlled release of IND for the potential treatment of chronic inflammatory diseases.

Using a spray drying method, Ozturk et al. prepared CS NP loaded dexketoprofen (dKTP) with high 73–84% EE and prolonged release at pH 6.8. The results of the in vivo anti-inflammatory activity tested by the HET-CAM assay showed that the system had good anti-inflammatory potential compared to free dKTP and identical to free dKTP at one-fifth the dose [130].

Spherical tristearin lipid NPs containing celecoxib (CLX) with a diameter of 188 nm and prepared by microemulsion technique allowed for the controlled release of CLX, so that 62% of the drug was released within 36 h. The formulation showed insignificant cytotoxicity in in vivo studies and, conversely, significantly (up to 88%) inhibited the formation of inflammation in an in vivo model of Complete Freund’s Adjuvant (CFA)-induced RA [131].

Badri et al. proposed a NP system suitable for the transdermal administration of IND for the treatment of inflammatory diseases while eliminating its side effects. The NanoDDS consisting of polycaprolactone prepared by precipitation had a particle size in the range of 220 to 245 nm, a zeta potential value of −19 to −13 mV, the EE was approx. 70%, and the drug content was 14% to 17%. An ex vivo study of the permeation of NPs through fresh human skin has shown that this formulation potentiates IND penetration through the skin [132]. Similarly, Yokota et al. prepared IND, KTP, and piroxicam NPs that were incorporated into hydrophilic ointments and compared their permeation through the skin and their ability to suppress acute carrageenan-induced rat paw inflammation in a rat model of chronic arthritis. The nanoDDSs showed significantly higher anti-inflammatory activity than the ointments with micronized NSAIDs and led to better overall healing [118].

Fenoprofen (FPF) encapsulated in a surfactant-based nanovesicular system (spanlastics) composed of Span 60 and Tween 60 (8:2 *w*/*w*) in the presence of Transcutol P as a transdermal enhancer [133] was tested for the topical administration of FPF while eliminating its gastrointestinal side effects. The EE of the system was 49.91 ± 2.60%, the particle size was 536.1 ± 17.14 nm, and approx. 61% of the drug was released through the cellulose model membrane after 24 h. Following topical application to carrageenan-induced rat paw edema, anti-edema activity (inhibition of inflammation) of this nanoDDS was found to be 3-fold higher than that of the bulk FPF in gel after 24 h, demonstrating that this system provides prolonged and increased anti-inflammatory activity in the treatment of arthritis [134].

A dual topical nanoDDS composed of bioceramic materials of calcium deficient hydroxyapatite (CDHA, Ca/P = 1.61) and tricalcium phosphate (CPH) for the treatment of bacterial infections and inflammation associated with periodontitis was prepared by microwave-accelerated wet chemical synthesis. The expected advantage was also bone-regenerative efficiency. CDHA was the carrier for the antibiotic tetracycline; CPH was the carrier for IBP. In vitro studies showed that the system was biocompatible with significant antibacterial and anti-inflammatory activity. In vivo implantation studies in rat cranial defects showed better bone healing and new bone formation at the end of week 12 compared to the control. Thus, this dual nanoDDS can be considered potentially suitable for the treatment of bone requiring multiple drug therapy [135].

A chlorine e6-labeled nanoDDS containing methotrexate (MTX) and human serum albumin (MTX@HSA) administered to mice with collagen-induced arthritis revealed the accumulation and longer retention of this system in inflamed joints, suggesting that the system has the ability to attenuate RA progression. At half the dose of MTX administered, the system had higher efficacy and lower systemic toxicity compared to free MTX [136]. For CS NP loaded MTX and DEX prepared by ion gelation, the controlled release of drugs in phosphate buffers at pH 7.4 and pH 5.8 was found. The activity was tested in vitro on HEK and RAW264.7 cells; the IC_50_ values of MTX NPs were 26.1 μg/mL on HEK and 7.7 μg/mL on RAW 264.7 cells; and the IC_50_ values of DEX NPs were 20.12 μg/mL on HEK and 7.37 μg/mL on RAW264.7. The increased uptake of the nanoDDS by RAW cells indicated the internalization of nanoDDS by phagocytosis. A higher in vivo anti-RA activity was observed after intraperitoneal injections compared to free/bulk MTX and DEX [137]. Combined nanoDDS containing MTX and AgNPs encapsulated in poly(ethylene glycol) (pegylated, PEG) PLGA nanospheres demonstrated pH-dependent drug release in an in vitro assay. The system without active compounds had an insignificant in vitro cytotoxic effect, while the whole system had a much greater effect on the viability of monocytes and macrophages than free MTX, a significant decrease in pro-inflammatory IL-1β, IL-6, and TNF-α was also found after inflammatory stimulation in vitro, suggesting promising potential for the use of combined NPs in the treatment of RA [138].

Aspasomes with MTX and the antioxidant ascorbyl palmitate with the particle size of 386.8 nm, ca. 19% EE, negative surface potential, and controlled constant drug release in vitro for 24 h were prepared for transdermal administration for the treatment of RA. The NanoDDS was blended into a hydrogel that was tested in vivo in a Wistar rat model of RA. The transdermal administration of MTX aspasome hydrogel after the 12^th^ day reduced rat paw diameter (21.25%), SGOT (40.43%), SGPT (54.75%), TNF-α (33.99%), IL-β (34.79%), cartilage damage (84.41%), inflammation (82.37%), panus formation (84.38%), and bone resorption (80.52%) compared to the control rats with arthritis and was halved better than the free MTX group, showing a positive effect of this RA system [96]. MTX was encapsulated in a fucoidan/CS polymer system in a 5:1 ratio. The size of the prepared NPs was in the range of 300–500 nm, zeta potential −45 mV, MTX content was approx. 14%, and EE was 80%. MTX in the nanoformulation did not affect the viability of fibroblasts and human keratinocytes and showed lower cytotoxicity than free MTX. Skin permeation studies showed that MTX in the nanoformulation passed through full-thickness pig ear skin and reached a 3.3-fold increase after 6 h compared to free MTX. The application of this nanoformulation has led to a significant reduction in pro-inflammatory cytokines produced by activated human monocytes, so that they can potentially be used for the topical administration of MTX to treat inflammatory skin-related diseases [139].

A combination of MTX and etanercept (the anti-TNF-α fusion protein, ~150 kDa, ETR) for targeted therapy could be a new possibility in psoriasis treatment. Lipid NPs fixed by a carbopol hydrogel were used for MTX and ETR co-delivery. A potential for drug transport into the skin affected by reduced transdermal permeation was studied. An in vitro experiment showed that MTX was released from lipid NPs sustainably for 8 h. NPs were identified as non-toxic toward fibroblasts and human keratinocytes. Compared to free MTX, an enhanced skin deposition of the methotrexate-lipid NP system was observed [140]. Targeted, sustained therapeutics delivery to human skin is restricted to lipophilic molecules having the molecular weight of <500 Da [133]. Such molecules are capable of crossing the stratum corneum. A novel thermoresponsive nanogel (tNG) encapsulating ETR was synthesized as a biocompatible protein carrier. No changes in its structure were observed and ETR was released from the tNGs due to temperature. Synthesis was performed without any organic solvents; the encapsulation of protein was made in situ during assembly. Application to inflammatory skin equivalents as well as tape striped human skin shown an efficient ETR delivery across the stratum corneum and into the viable epidermis, which proved efficient anti-inflammatory effects [141]. The enhancement of the transdermal delivery of ETR by microneedles was tested in both rabbits and rats. This was due to the fact that the transdermal delivery of the therapeutic is limited by restrictions on the size of DDS and also by hydrophobicity. It was shown that the microneedles significantly enhanced transport in vitro. This effect is possible by the transformation of the tight junction proteins beginning via binding of integrin to the nanotopography, continuing by phosphorylation of myosin light chain and activation of the actomyosin complex, which increased paracellular permeability [142].

Infliximab (IFX) is a chimeric monoclonal antibody blocking TNF-α. Its function is to induce clinical response and mucosal healing in IBD patients; however, its systemic administration evokes undesirable side effects. The oral delivery systems based on liposomes, aminoclay, which is a magnesium phyllosilicate functionalized with 3-aminopropyl groups and Eudragit^®^ (copolymer of methacrylic acid) were tested as orally administered antibody delivery systems. These carriers showed a minimal systemic exposure, narrow size distribution, and a high efficiency of encapsulation. A higher interaction between carriers and monocytes was observed than between carriers and lymphocytes in the peripheral blood mononuclear cells of IBD patients. Orally administered systems targeted to inflamed murine colitis led to the minimization of a systemic exposure. A significantly less colitis-induced body weight loss, colon shortening, and histomorphological score in tested mice were found in the case of all nanoDDSs consisting of IFX and these carriers than in the case of a group treated with dextran sulfate sodium (DSS)-induced colitis [143]. Polyesterurethane (PU) and its PEG form (PU-PEG) were also tested as IFX nanocarriers for the treatment of inflammation in an in-vitro epithelial model. The average size of the prepared NPs was 200–287 nm. After INF loading (INF-NPs), NPs size and zeta potential were increased, and no cytotoxicity was indicated. The uptake of PU NPs and cellular interaction proceeded similarly as in the case of polycaprolactone NPs; however, they were significantly higher than in the case of PLGA NPs. The rapid recovery of the epithelial barrier function in inflamed Caco-2 cell monolayers was achieved, and the levels of cytokine in inflamed monocytes decreased after applications of INF-PU and INF-PU-PEG NPs [144].

Adjunct therapy based on tolerogenic NPs is a perspective and widely applicable approach to prevent the antidrug antibodies (ADAs) formation against biologic therapies. A nanoDDS consisting of PLGA NPs bearing sirolimus (rapamycin) can induce persistent immunological tolerance to proteins that are co-administered in a treatment. It is characterized by a reduction in the activation of B cells, tolerogenic DCs induction, a regulatory T cell increase, germinal center formation, and the inhibition of antigen-specific hypersensitivity reactions. Intravenous co-administration of tolerogenic NPs with PEG-uricase prevented ADA formation in mice and non-human primates. It also regulated levels of serum uric acid in uricase-deficient mice. The subcutaneous co-administration of NPs bearing adalimumab ensued persistent ADA inhibition, which led to normalized anti-TNF-α antibody pharmacokinetics as well as to protection against arthritis in TNF-α transgenic mice [145].

Tacrolimus encapsulated in 212 nm lipidcore nanocapsules showed significantly higher inhibition of paw swelling after intraperitoneal administration in an in vivo study of arthritis induced by CFA [146].

The antihyperlipidemic drug fluvastatin (FVS) has been shown to have pleiotropic effects in RA in addition to its primary effect [147]. Therefore, FVS has been encapsulated in spanlastic nanovesicles (SNV) composed of Tween 80 or Brij 35 and either Span 60 or Span 80 for transdermal administration, which should reduce the overall burden of the body with this drug. Thus, spherical vesicles with a size of 201.54 ± 9.16 nm and EE of 71.28 ± 2.05% were prepared and gradually released approx. 90% of FVS in 8 h. A study in rats found an approx. 3–5-fold higher bioavailability of FVS from nanoDDS compared to oral solution and/or gel. In addition, there was a significant reduction in the expression of TNF-α, IL-10, and p38 MAPK [148].

Selected above-mentioned drugs and the properties of their nanoDDSs are summarized in Table 1.

### 4.3. NanoDDSs for Other Bioactive Agents

Gold and its compounds are known for their anti-inflammatory effects. De Araujo et al. [149] therefore prepared AgNPs using an environmentally friendly manner as potential nanoDDSs. The AgNPs were evaluated for their cytotoxic effect on HT-29 cells and also administered to male and female Swiss mice to assess their anti-inflammatory properties. Cell apoptosis was observed in dose-dependent concentrations ranging from 40 to 80 μg/mL. The best anti-inflammatory activity was observed at the dose of 1500 μg/kg, which reduced leukocyte migration by 49.3%.

NPs formed from PLGA with two molecular weights (high/low) and poly(dl-lactide) (PLG-L, PLG-H, and PLA, respectively) designed as non-biodegradable systems that target circulating inflammatory monocytes and neutrophils in the vasculature to prevent them from migrating to inflamed tissue were investigated for their association with monocytes and neutrophils and their overall effect on the course of inflammation. The particles were administered intravenously to mice with experimental autoimmune encephalomyelitis (EAE). After six days of administration, individuals with PLG-H were found to have significantly lower manifestations of EAE compared to the control and the mice treated with PLG-L and PLA NPs. In vivo and in vitro experiments showed that PLG-H had a high association with immune cells with a preferential association with blood neutrophils. PLG-H also retained immune cells from the central nervous system with increased accumulation in the spleen. Thus, it was confirmed that particle composition affects association with inflammatory monocytes and neutrophils in the vasculature, with the potential to redirect trade and alleviate inflammation [86].

Fluorescent CS-, PEG-, and non-functional PLGA micro- and nanoparticles with mean hydrodynamic diameters of 3000 nm and 300 nm were prepared by solvent evaporation techniques as targeted DDSs that selectively accumulate in inflamed mucosal areas without systemic absorption. Using ex vivo experiments and histomorphological and electrophysiological studies of inflamed mucosal tissues, it was observed that NPs showed increased translocation and deposition compared to microparticles in healthy and inflamed mucosa. CS-functionalized particles adhered to the tissue surface, showing the lowest translocation and deposition of particles in healthy and inflamed tissues. PEG-functionalized nanoparticles showed the highest translocation of healthy (2.31%) and inflamed mucosa (5.27%). Based on the results, the nanosystem can be considered potentially suitable for innovative drug delivery for IBD treatment [150].

Acetate gum (GA) stabilized hesperidin (HP) coated AgNPs were tested in the CFA induced arthritis model. HP-GA-AgNPs showed mild to moderate tissue swelling, and reduced degenerative changes along with mild articular changes. Histology showed less influx of inflammatory cells and reduced granulomatous inflammation in ankle tissues in the presence of HP-containing GA-AgNPs [151]. Rao [152] published a study describing that routine-stabilized AgNPs inhibited the production of proinflammatory agents such as IL-6 and TNF-α in a model of RA disease induced by CFA in Wistar rats, making the system a potential RA therapeutic.

**Figure 5:**
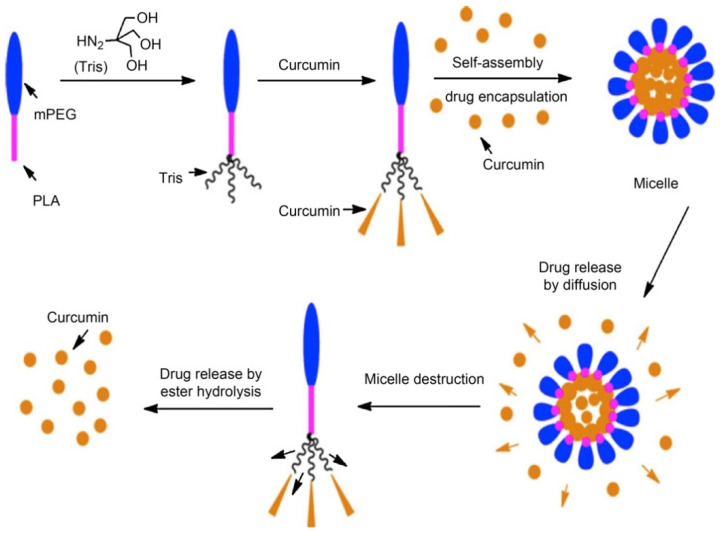
Methoxy-PEG–PLA–Tris–CUR nanoDDs: synthesis and loading with a consequent release of CUR. Reproduced with permission from [156], Elsevier, 2014.

Curcumin (1,7-bis(4-hydroxy-3-methoxyphenyl)-1,6-heptadiene-3,5-dione, CUR) is a natural polyphenol found in *Curcuma* spp. *Curcuma longa* is traditionally used as an medicinal plant in Asian countries, and CUR extracted from the rhizome is widely used as a spice. It is used as a colorant (code E 100) in the food industry. CUR has strong antioxidant, anti-inflammatory, antimutagenic, antimicrobial, and anticancer effects, so it is widely used in folk medicine. Its weaknesses are low water solubility, low absorption from the gastrointestinal tract, poor stability in body fluids, rapid metabolism, and rapid clearance. For these reasons, its therapeutic use in medicine is limited, but great efforts have been made for a long time to investigate suitable usable nanoformulations [153,154,155]. Due to the huge number of such described nanoformulations (e.g., Figure 5), the overview of which would require a separate paper, only a few reviews or recently published important articles are listed here [78,156,157,158,159,160,161,162,163,164]. For example, Coradini et al. [165] formulated CUR and resveratrol into lipid core nanocapsules. The whole system was administered intraperitoneally (1.75 mg/kg) for eight days twice per day to rats with CFA-induced arthritis. Both nanoformulated polyphenols inhibited paw swelling by 37–55% compared to the same dose of bulk substances. In addition, treatment was observed to minimize histological changes such as synovial fibrosis, cartilage, and bone loss.

Tetrahydrocurcumin (tCUR), a stable colorless hydrogenated product of CUR with good antioxidant and anti-inflammatory properties, was incorporated into SLNs prepared by a microemulsification technique. NPs were ellipsoidal in shape with the mean particle size of 96.6 nm and the zeta potential of −22 mV. The total drug content and tCUR-SLN capture efficiency were 94.51 ± 2.15% and 69.56 ± 1.35%, respectively. The system was incorporated into the hydrogel. Permeation studies showed approx. 17-fold higher skin permeation of tCUR-SLNs in the gel than free tCUR in the gel. The formulation was found to be non-irritating and to increase the anti-inflammatory activity of tCUR, suggesting the possibility of using these nanoDDSs with inflammatory skin diseases [166].

[6]-Shogaol formed in ginger during drying or cooking has significant anti-inflammatory effects, but is metabolized extremely rapidly after oral administration. Therefore, Yang et al. encapsulated [6]-shogaol in the NPs of natural lipids, which protects it while allowing controlled release into the intended target of the drug in the colon. After oral administration, this nanoDDS showed excellent efficacy compared to free [6]-shogaol in a mouse model of DSS-induced colitis. Released [6]-shogaol was metabolized to active metabolites that downregulated the pro-inflammatory factors TNF-α, IL-1β, and IL-6 and upregulated the anti-inflammatory factor IL-10 in inflamed Raw 264.7 cells. Wound healing tests confirmed that the metabolites also accelerated the wound repair process of Caco-2 cells at the concentrations observed in the colon (1.0 μg/mL) [167].

The biocompatible copolymer of methoxy-poly(ethylene glycol) (mPEG)-PLGA with encapsulated benzoylaconitine (BAC) isolated from *Aconitum kusnezoffii* Reichb showed compatibility for activated macrophages and good compatibility with red blood cells. In addition, the bioavailability of BAC increased. The system reduced TNF-α and IL-1β secretion by 70% and 66%, respectively, compared to activated macrophages, and also overexpression of NF-κB p65 [168].

Triptolide (TP), a diterpenoid epoxide produced by *Tripterygium wilfordii*, has anti-inflammatory effects and is able to protect cartilage in RA. Unfortunately, it is also immunosuppressive and highly toxic [169]. To reduce toxicity, a nanoDDS consisting of poly-γ-glutamic acid-grafted di-*tert*-butyl l-aspartate hydrochloride (PAT) with a diameter of 79 ± 18 nm, PDI 0.18, zeta potential −32 mV, drug 48.6% EE, and filling capacity of 19.2% was prepared by Zhang et al. In vitro assays showed reduced toxicity and apoptosis induced by free TP on RAW264.7 cells. The evaluated system of LPS/INFγ-induced cytokine expression of macrophage and in vivo PAT accumulated in inflammatory joints. In addition, PAT reduced inflammatory synovial tissue area, cartilage loss, tartrate-resistant acid phosphatase-resistant osteoclast area, and bone erosion in both the knee and ankle joints and showed a similar beneficial effect as free TP [170].

DCs can be considered as “specialized sensors” of the first line of foreign materials invading the organism. On the contrary, their inappropriate activation contributes to inflammatory diseases and immunopathologies [171]. Deng et al. [172] prepared NPs targeting DC for the regulation of intestinal immune homeostasis. Orally administered nanoparticles prepared from broccoli extracts protected C57BL/6(B6) mice from DSS-induced colitis by activating adenosine monophosphate-activated protein kinase in DC.

Hybrids of peptides and gold nanoparticles (pept-GNPs) were prepared, which act as potent nanoinhibitors of Toll-like receptor 4 (TLR4) signaling by modulating the endosomal acidification process. TLR4 is a transmembrane protein, the activation of which leads to the intracellular NF-κB signaling pathway and the production of inflammatory cytokines responsible for activating the innate immune system. Lipopolysaccharide (LPS) is its best known ligand [173]. It was found that NP size significantly affects TLR4 inhibition; pept-GNPs with a 20 nm gold core showed the strongest activity against TLR4 in the subsequent production inhibition of cytokines compared to a gold core of 13 nm or 5 nm. These in vitro results were confirmed by the in vivo experiment performed on a mouse model of LPS-induced inflammation. This size of NPs had higher cellular uptake and stronger endosomal pH buffering capacity, which contributed to its increased therapeutic effects in reducing TLR4 activation in vitro and in vivo, which suggests that these nano-therapeutics are promising for the treatment of acute and chronic inflammatory diseases [174].

An mRNA gene therapy is potentially promising for the treatment of inflammatory diseases. However, mRNA is a very labile molecule and its practical use requires a suitable carrier, in which the mRNA can be transported [171]. A system consisting of microRNA-29 (mR-29) and supercarbonate apatite nanoparticles (sCA) was prepared. Injection into the tail veins of mice prevented DSS-induced inflammation. The nanosystem was found to inhibit the interferon-associated inflammatory cascade. Subcutaneous administration also inhibited inflammation and targeted CD11c and DCs in the inflamed mucosa, where IL-6, IL-23, and TGF-β production was suppressed. The study suggests that sCA-mR-29 represents a potentially promising pathway in the development of nucleic acid-based IBD therapeutics [175,176]. Prepared lipid nanoparticles (LNPs) were composed of cationic lipid, neutral lipid of 1,2-dioleoyl-sn-glycero-3-phosphoethanolamine (DOPE), and *N*-(carbonyl-methoxy-PEG 2000)-1,2-dimyristoyl-sn-glycero-3-phosphoethanolamine sodium salt (PEG2000-DMPE). siRNA [171] was encapsulated in the matrix and the system had an in vitro knock-down effect on mouse inflammatory peritoneal macrophages and DCs.

## 5. Conclusions

Although some progress has been made in recent years in the design of new, especially high-molecular-weight drugs for the treatment of chronic inflammatory diseases, these diseases, especially arthritis, inflammatory bowel diseases, and various other autoinflammatory diseases, represent a significant reduction in patient quality of life and pressure on health and social care. Some cytostatics and antifungal drugs in nanoDDSs were approved and successfully introduced into clinical practice. Therefore, it is hoped that nanotherapy will also become a promising approach in the treatment of chronic inflammatory diseases. However, there is a fundamental difference between short-term therapy with anti-invasive drugs (which may generally demonstrate higher toxicity and overall burden on the body) and long-term treatment of chronic diseases (that must mean minimal burden on the body, which remains a major challenge for all nanoformulation designers). Individual studies focus on the increasingly sophisticated preparation of nanoDDSs, the in vitro evaluation of the cytotoxicity of entire nanoformulations, and the in vitro/in vivo inhibition of inflammatory responses by reducing the synthesis/secretion of pro-inflammatory mediators. Some studies that have addressed the transdermal administration or inflammation of the mucous membranes have also investigated the local irritability of nanoformulations and the effect on the healing of the affected mucous membranes. In the future, it would be ideal to combine both an anti-inflammatory effect (and an anti-infective effect in cases of primary infection or secondary attachment of infection to damaged tissue) with an increased potential for mucosal/skin healing. As such systems are intended for long-term therapy, to enable the registration of nanotherapeutics by the relevant authorities, it is extremely important to ensure their safety after administration (i.e., to know maximum about the toxicological effect in the body and in the environment after excretion from the body as well as the pharmacokinetics of entire nanoDDSs) and develop the simple large-scale production of nanotherapeutics, which would reduce the production costs.

## Figures and Tables

**Table 1:** Summary of selected drugs with anti-inflammatory effect and their nanoformulations.

Active Agents	Carrier	NPs Size (nm)	Loading Efficiency (μg/g) or EE (%)	Release/Stability	Activity/Efficiency	Ref.
DEX + OVA	PLGA	864	0.978 ± 0.022 DEX 148.37 ± 10.92 OVA	release kinetics similar at pH 3.0 and pH 7.0; after 2 h of incubation at pH 3.0, 83% DEX and 77% OVA remained in DDS, respectively	tolerogenic properties	[119]
DEX	ascorbyl palmitate	1–2 μm fiber diameter, 20–50 μm length	5 and 10 mg/mL	stable for 16 days (PBS, 37 °C), addition of esterase and lipase induced rapid, dose-dependent release	negatively charged surface preferentially adhere to positively charged artificial surfaces, IBD treatment	[120]
DEX + diclofenac	polylactide	230	EE 66%	stable for 7 days at pH 7.4; hydrolyzed by esterase within 1.5 h	twin-drug	[121]
budesonide	BSA	225	–	stable for 28 days at room temperature	IBD treatment	[122]
methyl-prednisolone	BSA	131	EE 71%	–	treatment of membranous glomerulonephritis	[123]
prednisolone acetate	CS, SD, PVA	321–976	EE 36–58%	–	ophthalmic gel	[124]
diclofenac, IBP, etodolac, paracetamol	NCC	3–5	EE 36–61%	drug release in 120–180 min	sustained release formulations of NSAID	[125]
IBP KTP NBT	Capmul^®^, Gelucire^®^	161 136 152	EE 52% EE 74% EE 68%	sustained release of IBP for 6 days, controlled release of NBT up to 48 h, release of KTP for 4 days	sustained release formulations of three NSAIDs	[126]
IND KTP	stearic acid	274 254	EE 82% EE 71%	ca. 60% released due to the burst effect, further release due to continuum effect	topical/oral/nasal formulations	[127]
NPX	lipid carriers	279–591	EE 99%	stable for 1 year at 25 °C, sustained release >7 days	treatment of inflammation of the temporomandibular joint and peripheral tissues	[128]
IND	CS, liposomes	431–593	EE 98%	stability 3–6 weeks; 61–76% released at pH 6.8 after 270 min, 71–77% released after 1410 min	stable and highly loaded nanoDDSs	[129]
dKTP	CS	726–780	EE 73–84%	81–93% of released after 48 h	novel nanoDDS for anti-inflammatory therapy	[130]
CLX	tristearin	188	loading efficiency 93%	sustained drug release of 62% for 36 h	enhanced bioavailability and therapeutic efficiency	[131]
IND	polycaprolactone	220–245	EE 70–76%	no significant changes in 30 days	topical application to reduce side effects and frequency of administration	[132]
IND KTP piroxicam	hydroxypropyl cellulose	78 IND 74 KTP 81 piroxicam	–	–	semi-solid nanoDDSs	[118]
FPF	Tween 60: Span 60 (2:8), Transcutol P	536	EE 49%	61% FPF released after 24 h	improving topical delivery	[133]
FPF	spanlastic gel	208–837	EE 49%	no change at 4–8 °C for 6 months	improving topical delivery	[134]
MTX	HSA	30–116	loading efficiency 87%	MTX released faster in acidic conditions	increased therapeutic effect, insignificant systemic effects	[136]
MTX DEX	CS	217 329	EE 55% EE 10%	release at pH 5.8 and 7.4 due to burst effect within 5 h; then high release only at pH 5.8	high biocompatibility, increased therapeutic efficacy, reduced toxicity	[137]
MTX	ascorbyl palmitate	386–650	EE 31–90%	release varied from 76% to 95% over 24 h	topical delivery	[96]
MTX	fucoidan:CS (5:1)	306–475	EE 79–95%	no significant changes over 6 weeks	treatment of skin-related inflammations	[139]
MTX + ETR	cetyl palmitate, Tween 80	356	EE 88%	no significant changes over 8 weeks at 25 °C	psoriasis treatment	[140]
ETR	polyglycerol, poly(*N*-isopropylacrylamide)	155	–	released 82% after 48 h	psoriasis treatment	[141]
ETR	MNA	–	–	–	longer lasting transdermal DDS	[142]
IFX	BSA, aminoclay, Eudragit^®^	426	EE 28%	no significant degradation	IBD treatment	[143]
IFX	PU-PEG	1537–1706	EE 48–51%	–	IBD treatment	[144]
sirolimus	PLGA	–	–	–	tolerogenic dendritic cell induction, regulatory T cell increase, B cell activation reduction, germinal center formation	[145]
FVS	Span 60/ Span 80, Tween 80/ Brij 35	167–692	EE 34–80%	released 67–92% after 8 h	pleiotropic effects in RA	[148]
